# Additively Manufactured Density-Graded Dual-Material Auxetic Structures: Enhanced Energy Absorption and Shape Recovery

**DOI:** 10.3390/mi17050570

**Published:** 2026-05-03

**Authors:** Mohammad Faisal Ahmed, Kyle Primes

**Affiliations:** Department of Industrial and Engineering Technology, Southeastern Louisiana University, 801 N. Oak Street, Hammond, LA 70402, USA

**Keywords:** auxetic, dual-material, density gradient, energy absorption, in-plane compression, negative Poisson’s ratio

## Abstract

The auxetic reentrant structure, one of the most widely studied negative Poisson’s ratio structures for its geometric simplicity, has long seen limited applications due to challenges emanating from its inherent design when built from a single rigid or flexible material. This paper aims to address these challenges by taking advantage of dual-material extrusion technology and density gradient design strategy. Two density gradient reentrant auxetic structures are proposed and fabricated using material extrusion additive manufacturing in single-material (flexible) and dual-material (rigid/flexible) modes, with the introduction of a novel dual-material interface design. In-plane compression tests are carried out to assess the energy absorption characteristics of the structures. The results show that dual-material structures exhibit higher yield stress, mean crushing force, peak crushing force, and maximum crushing force, as well as superior specific energy, energy dissipation, and energy release compared to single-material structures. Dual-material structures also demonstrate high lateral stiffness, minimizing elastic instability, a highly desirable feature for reusable energy-absorbing structures with high shape recovery capability. The results substantiate the significance of the synergy between the dual-material and density gradient designs proposed in this study. Overall, the key findings of the study may serve as a reliable reference for the design of future lightweight energy-absorbing structures.

## 1. Introduction

Auxetic behavior, or a negative Poisson’s ratio effect, is demonstrated by a class of materials with counterintuitive deformation characteristics, where the materials expand laterally when they are stretched and contract when they are contracted [1,2]. Owing to this unique characteristic, auxetic materials are reported to have high shear resistance, fracture toughness, indentation resistance, and energy absorption capabilities. Additionally, they exhibit synclastic behavior and variable permeability, unlike their conventional positive Poisson’s ratio counterparts [3,4,5,6,7,8]. Due to these superior properties, they are potential candidates for a wide range of practical applications, including, but not limited to, biomedical instruments [9,10], smart filters [2,11], aerospace components [12], prostheses [13,14], sporting goods [15,16], smart sensors [17], dampers [18,19], and non-pneumatic tires [20]. Consequently, the recent widespread adoption of additive manufacturing technologies has spurred the rapid exploration of a variety of cell geometries and deformation mechanics for developing auxetic structures or mechanical metamaterials (i.e., architected structures) with improved performance.

Gradient design strategy has attracted significant attention in the mechanical metamaterials or architected structure research community as it enables strategic incorporation of global or local density gradients towards enhanced mechanical performance [21,22]. Accordingly, researchers have explored different design methodologies that go beyond a regular hexagonal honeycomb structure. For instance, Shao et al. investigated unidirectionally graded auxetic reentrant honeycomb and found that negative gradient auxetic honeycomb dissipates energy better than positive gradient auxetic honeycomb under medium and high compression velocities [23]. Staffova et al. used a biaxial density gradient on reentrant auxetic honeycomb and found that under quasi-static compression, the specific energy absorption of the biaxial centric gradient structure surpassed that of the uniform density structure by as much as 241% [24]. Wang et al. also found that the specific energy absorption of reentrant arc-curved honeycomb increased by 11% and 16.8% for thickness gradient and arc angle gradient, respectively [25]. Research studies on auxetic structures other than the traditional reentrant design (e.g., star, tetra-petal, hexachiral, peanut, circular) showed that the gradient design strategy can be used as an effective method to improve energy absorption capacity [26,27,28,29,30,31].

Due to the rapid advancements in multi-material extrusion technologies, researchers have explored a variety of cellular architecture designs and their associated deformation mechanics with the goal of overcoming the trade-offs that are commonly required when limited to single-material structures. Using a hard-shell and soft-core design for a conventional hexagonal structure, Yavas et al. found that the energy absorption capacity of dual-material lattices can be significantly greater (by about 2–3 times) than that of the individual constituents [32]. Mao et al. studied hierarchical soft/hard dual-material honeycomb and reported that a dual-material design exhibits excellent synergy in enhancing the energy absorption performance compared to a single-material design [33]. Similarly, the benefits of using a dual-material design for auxetic structures have also been reported. For instance, with strategic placement of elastic material in the joint regions and stiff material in the beam/wall regions, Wang et al. found that dual-material reentrant auxetic metamaterials deform without the beam or wall buckling, an issue that is inevitable in single-material designs [34]. Bodaghi et al. used 4D printing to study reentrant auxetic meta-structures made from soft and hard components. They found that the mechanically induced plastic deformation and energy dissipation process are fully reversible with heat [35]. By introducing a lower stiffness material in the hinge/corner area of reentrant and anti-tetrachiral auxetic structures, Johnston and Kazanci found that dual-material structures perform better in multiple loading cycles due to the compression occurring through elastic buckling in comparison to plastic buckling in single-material structures [36]. Gunaydin et al., on the other hand, introduced carbon- and glass-fiber-reinforced nylon in the vertical ligaments of a dual-material reentrant auxetic structure, and reported 60%, 104%, and 201% enhancement in specific energy absorption, compressive strength, and modulus, respectively, in comparison to single-material nylon structure [37]. To achieve enhanced structural stiffness and retain auxetic behavior under compressive load, Su et al. proposed a dual-material reentrant structure with additional soft arch-like beams and hinges. The authors found that the typical buckling issue of the beam/wall can be significantly reduced if the strength of the hinge is kept small, thereby minimizing the reduction in auxetic behavior due to increased stiffness [38]. However, as this approach compromises structural stiffness, Dong et al. introduced a curved reentrant auxetic structure using a rigid–soft–rigid sandwich structure and found that soft thermoplastic polyurethane (TPU) effectively alleviates stress concentration and exhibits higher resilience, enabling the structure to withstand multiple loads [39]. More recently, Zhang et al. introduced polyimide (PA) into a carbon fiber/polyimide (CF/PA) reentrant structure as an interlocking hinge that could effectively overcome the stress concentration at the hinge region, preventing fracture damage caused by the high stiffness of CF/PA [40].

Due to the inherent structural design of the inward-facing ligaments (beams or walls) of reentrant auxetic metamaterials, the rotation and bending deformation of hinges (joints) and ligaments govern the stiffness and Poisson’s ratio. As such, the stiffness of the reentrant honeycomb is lower than that of its traditional honeycomb counterparts, limiting its structural applications [38,41]. To achieve higher stiffness, the beam or wall require a higher stiffness material, whereas the hinge, on the other hand, requires a low stiffness material to promote flexibility [34]. This underlying contradiction exists for single-material reentrant auxetic structures made from either rigid or soft polymers. When rigid polymer is used, the structure demonstrates high stiffness but undergoes plastic deformation upon collapse [36,39]. While a reentrant auxetic structure made from soft material can potentially excel in cyclic loading applications due to its partial or full shape recovery capabilities, it experiences global instability under compressive load, causing it to buckle laterally and deviate from symmetric to asymmetric collapse, due to the lack of lateral stiffness [42,43,44]. Therefore, the structure can no longer reach full densification through inward collapse, an outcome ideally expected from a reentrant auxetic structure, attributed to its high indentation resistance and energy absorption properties [3,4,18]. The existence of these challenges necessitates further design exploration of reentrant auxetic structures, with the possible synergy between density gradient and dual-material printing strategies.

In this study, a new reentrant auxetic structure is proposed leveraging on gradient design methodology and a dual-material extrusion system, with the goal of achieving high energy absorption, shape recovery, and reusability. Two density gradient reentrant auxetic structures were designed by incorporating thickness gradient, as well as their uniform density counterpart of similar relative density. These were fabricated in single-material (flexible) and dual-material (rigid/flexible) modes using the fused filament fabrication material extrusion method. The in-plane quasi-static compressive behavior was investigated using experimental and finite element simulation methods. A comparative study of the reentrant auxetic structures was also carried out to determine the effects of the gradient strategy and print modes (single-material vs. dual-material) on the mechanical performance and deformation patterns.

## 2. Methodology

### 2.1. Materials

Polyflex^TM^ TPU95 and PolyMax^TM^ PC 3D printing filaments were purchased from Polymaker LLC, Missouri City, TX, USA. The densities of TPU and PC are 1.15 g/cm^3^ and 1.19 g/cm^3^, respectively. The diameter of the filaments is 2.85 mm. TPU95 has a Shore hardness of 95A. It can elongate more than three times its original length.

### 2.2. Design Method for Auxetic Structures

#### 2.2.1. Dual-Material Structures

**Figure 1:**
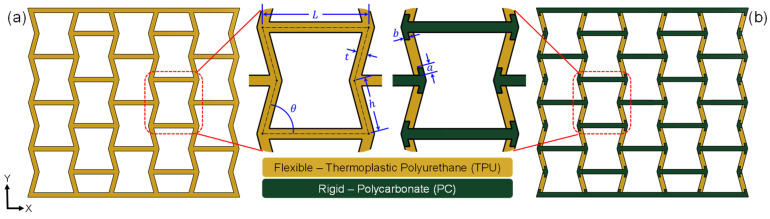
Design parameters used for reentrant (**a**) flexible single-material (SM) unit cell and (**b**) rigid/flexible dual-material unit cell.

In this study, all auxetic structures were designed using the well-known reentrant honeycomb unit cell as shown in Figure 1. The single-material (SM) structure was designed for printing with flexible TPU, whereas the dual-material (DM) structure was designed for printing with flexible TPU (yellow regions) and rigid PC (green regions). Typical values adopted for the significant geometrical features of the structures are listed in Table 1. For the DM structure, flexible TPU was placed along the inclined struts and rigid PC was placed along the horizontal struts so that TPU can offer extensive deformation and PC can deliver stability to the structure. While there are different methods used to design joint interfaces for multi-material prints [45,46], an arrowhead design was adopted in this study for two reasons: (1) to prevent premature failure in the interface region during compression loading and (2) to generate higher reactionary force during unloading. Two additional design parameters associated with the arrowhead design were introduced. The dual-material interface across the inclined struts was set as constant (a=1 mm) for all structures, whereas the interface across the horizontal axis was variable, and was half of the strut wall thickness (b=t/2).

#### 2.2.2. Density-Graded Structures

**Figure 2:**
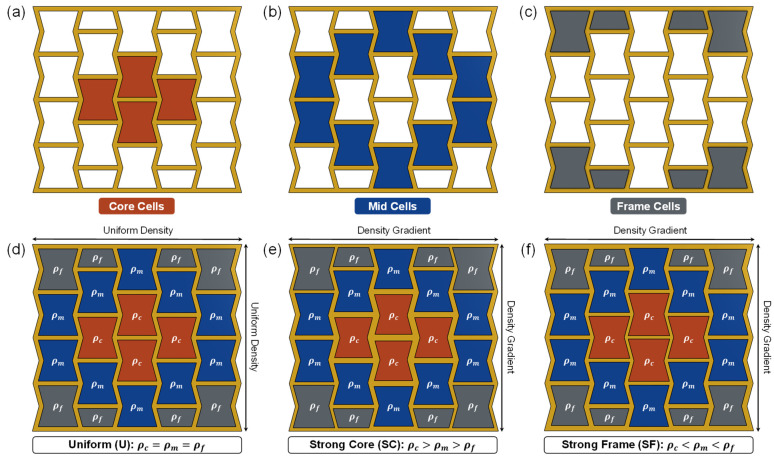
Proposed density gradient strategy using (**a**) core cells, (**b**) mid cells, and (**c**) frame cells to generate (**d**) uniform, (**e**) strong core, and (**f**) strong frame auxetic structures.

A density gradient was introduced by categorizing the unit cells into three cell groups: (1) core cells, (2) mid cells, and (3) frame cells (Figure 2). The four centermost adjacent cells were identified as core cells. The cells directly adjacent to the core cells were identified as mid cells and the cells directly adjacent to the mid cells were identified as frame cells. The densities of these cells were annotated as $\rho_{c}$, $\rho_{m}$, and $\rho_{f}$, respectively. Using these cell groups, two density-graded structures were designed to compare with the uniform density structure, one with a strong core (SC) and the other with a strong frame (SF). For the strong core design, the density decreased from the center to the outermost wall ($\rho_{c}>\rho_{m}>\rho_{f}$), and vice versa in the case of the strong frame design ($\rho_{c}<\rho_{m}<\rho_{f}$). Though this study used a 4 cells × 5 cells auxetic structure, the density gradient methodology adopted herein can be scaled up to accommodate any structure that is larger in size.

**Figure 3:**
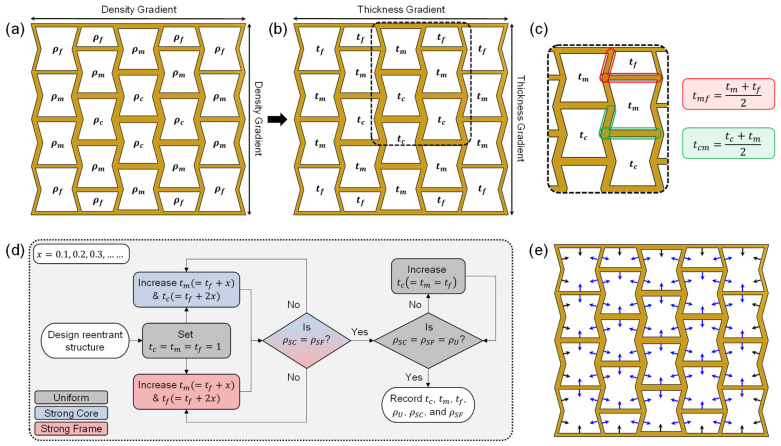
Gradient methodology: (**a**) density gradient, (**b**) thickness gradient, (**c**) definition of shared wall thickness, (**d**) iterative design method, and (**e**) thickness directions (black arrows indicate thickness direction for unshared walls and blue arrows for shared walls).

**Figure 4:**
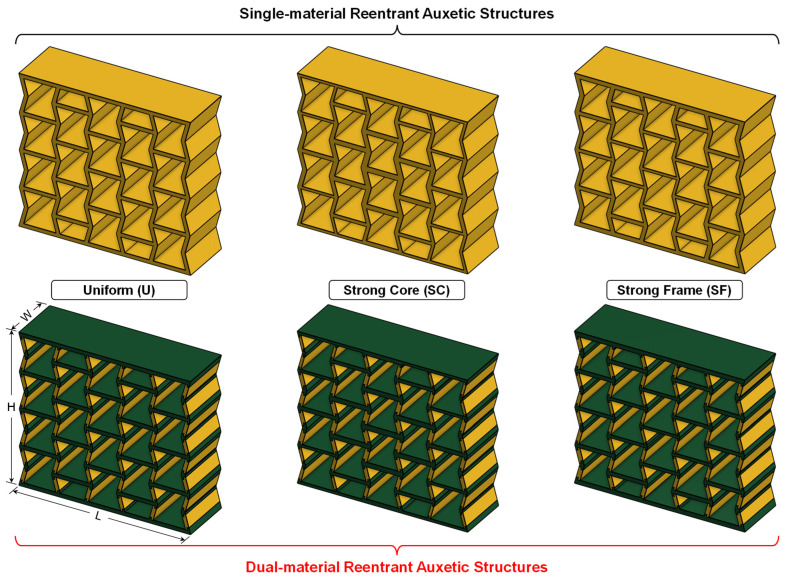
Length, width, and height annotations for single-material (SM) and dual-material (DM) structures.

The proposed density gradation was introduced by grading cell wall thicknesses ($t_{c}$, $t_{m}$, and $t_{f}$) as shown in Figure 3b. It should be noted that there are two types of walls. These should be identified separately to achieve density-graded auxetic structures for two important reasons: (1) to prevent the formation of any cone on the outer columns along the Y axis [47,48] and (2) to prevent any cell inside the structure from significantly deviating (e.g., stretched or compressed) from the conventional reentrant shape [49]. These two types of cell walls are identified as (1) shared walls and (2) unshared walls. A shared wall is a cell wall that is shared between two adjacent cells, and an unshared wall is a cell wall that belongs to only one cell. The thickness of the shared cell walls was defined as the average of the two cell wall thicknesses of the adjacent cells (as shown in Figure 3c). The thickness of the unshared walls was equal to the corresponding cell wall thickness. Since the number of core, mid, and frame cells is not equal, an iterative design method (Figure 3d) was implemented to ensure comparable experimental conditions without compromising relative density. First, all three specimens were designed using a minimum wall thickness (=1 mm) restricted by the print resolution. Next, the wall thickness for the SC and SF was increased in steps to match their densities. After that, the wall thickness of the uniform structure was increased to match this density. Finally, these numbers were recorded for comparison purposes. Figure 3e shows the process of increasing the wall thickness in relation to Figure 3c,d for shared (blue arrows) and unshared (black arrows) walls. Based on these considerations, six specimens were designed, three for single-material (SM) and three for corresponding dual-material (DM) designs (Figure 4). Table 2 summarizes the design parameters, geometric specifications, and theoretical relative densities ($\rho_{r}$) of the auxetic structures. The theoretical relative density was defined as the ratio of specimen density (ρ) to solid density ($\rho_{s}$) that constitutes the specimen wall.

### 2.3. Fabrication of Density-Graded Auxetic Structures

All auxetic structures were fabricated using a fused filament fabrication (FFF) material extrusion-based 3D printer (LulzBot Taz Pro, Fargo Additive Manufacturing Equipment 3D, LLC, Fargo, ND, USA). The printer supports printing with a flexible filament such as TPU, as well as with a high-temperature filament such as PC. Cura LulzBot Edition 4.13 was used as the slicing software for 3D printing of all the single-material and dual-material samples. The *Z*-axis was used as the build direction to print the samples. The default print parameters for the “High Detail” profile were used. The associated print process parameters are listed in Table 3. All the default parameters were kept unchanged except combing mode. Combing mode was set to “on” to prevent stringing [22]. Three replicates for each specimen group were printed, and the dimensional properties, mass, and relative density of all six specimen groups are listed in Table 4.

### 2.4. In-Plane Uniaxial Quasi-Static Compression Test

In-plane uniaxial compression tests were carried out to (1) assess the stress–strain profiles, (2) analyze force–displacement data for extracting mechanical properties and energy absorption characteristics, (3) evaluate the deformation behavior, and (4) identify the shape recovery and durability of the structures.

Compression tests were carried out using a universal testing machine (Mark-10 F1505S-IM, Mark-10 Corporation, Copiague, NY, USA), equipped with a 7.5 kN load cell. All samples were compressed up to 50% compressive strain in the Y direction under displacement control with a constant strain rate of 0.00167 s^−1^ (i.e., 10% strain/min as per ASTM D1621-16 [50]) and then unloaded at the same rate. The loading and unloading cycle was repeated 5 times for each sample. Each sample was positioned on the fixed bottom platform of the machine, and the top platform was used to apply the displacement loading on the top surface of the sample. The computer software used to control the machine automatically recorded the force–displacement data. Nominal stress was obtained by dividing the recorded force by the specimen’s original cross-sectional area, while nominal strain was determined based on the change in deflection at the interface of the sample with the platforms. The deformation process was captured at 30 fps using a high-resolution mirrorless digital camera (Canon EOS M50 Mark II, Canon Incorporation, Tokyo, Japan) equipped with an EF-M 15–45 mm f/3.5–6.3 IS STM zoom lens. The recorded videos were analyzed using the video analysis software KINOVEA^®^ v.2023.1.2 (Kinovea Open-source Project, www.kinovea.org).

### 2.5. Mechanical Characterization

The experimental elastic modulus and yield stress were identified from stress–strain curves according to ASTM D1621-16 [50]. The mean crushing force ($F_{mean}$) represents the average value of the load during the compression, which can reflect the energy absorption capacity per unit displacement as follows:

(1) $$F_{mean}=\frac{EA}{d}$$

where d refers to displacement and EA refers to energy absorption, which can be obtained from the area under the force–displacement curve of the loading cycle as follows:

(2) $$EA=\int_{0}^{d}F(x)dx$$

The peak crushing force ($F_{peak}$) is the peak force in the force–displacement curve during the initial stage of compression. A cellular structure that demonstrates a flat stress plateau with minimum oscillations is preferred for energy absorption applications. The crushing force efficiency (CFE) signifies the consistency of the structural load and can be obtained as follows:

(3) $$CFE=\frac{F_{mean}}{F_{peak}}$$

In addition to CFE, load uniformity (LU) can be used to evaluate the consistency of the structural load when $F_{peak}$ is not present. This is achieved by using the maximum crushing force ($F_{max}$) in the denominator instead, as follows [51,52]:

(4) $$LU=\frac{F_{mean}}{F_{max}}$$

The volumetric energy absorption capacity (also known as specific energy absorption, ${SEA}_{v}$) is quantified as the area under the compressive stress–strain curves, and provides an indicator of the energy absorbed per unit volume [53]:

(5) $${SEA}_{v}=\int_{0}^{\varepsilon }\sigma (\varepsilon )d\varepsilon$$

The gravimetric energy absorption capacity (also known as specific energy absorption, ${SEA}_{m}$), provides an indicator of the energy absorbed per unit mass and is quantified as

(6) $${SEA}_{m}=\frac{W_{v}}{\rho }$$

The hysteresis loop in the stress–strain profile represents the dissipated energy ($E_{d}$) of the reentrant structures, the value of which can be calculated as follows:

(7) $$E_{d}=EA-E_{r}$$

where $E_{r}$ is the energy released during the unloading cycle. A large value for $E_{d}$ and $E_{r}$ indicates better damping and recovery capabilities, respectively.

The Poisson’s ratio of the structures was identified by analyzing the specific frames from the recorded deformation process. The strain in the X direction was estimated from the deformation across the middle plane of the structure, and the strain in the Y direction was estimated from the timestamp of the corresponding frame. Finally, the Poisson’s ratio was calculated as follows:

(8) $$\nu =-\frac{\varepsilon_{x}}{\varepsilon_{y}}$$

where $\varepsilon_{x}$ and $\varepsilon_{y}$ are transverse and longitudinal strains, respectively.

Recovery ratio refers to the ability of the structure to recover to its original shape. It is the ratio of the recovered displacement after unloading to the applied displacement. All the aforementioned properties were obtained for each replicate tested for each specimen group, and the corresponding average and standard deviations were reported.

### 2.6. Finite Element Analysis

**Figure 5:**
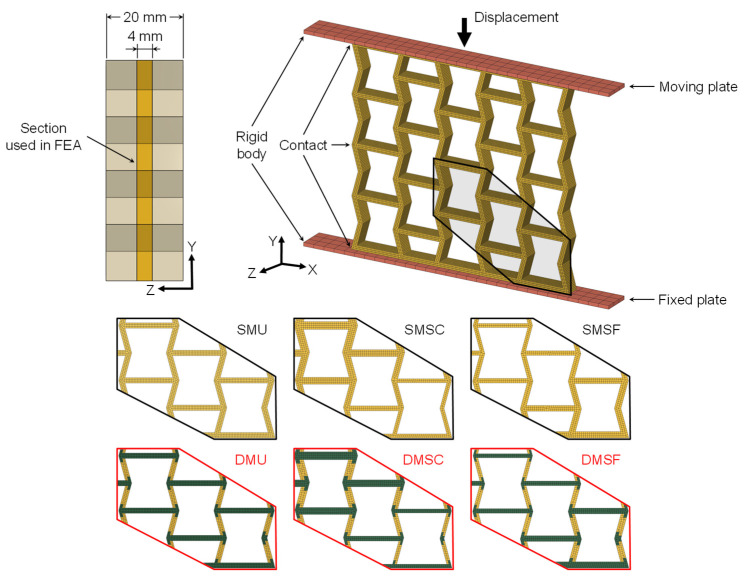
Boundary conditions used for finite element analysis.

The compressive deformation behavior of the reentrant auxetic structures was simulated using finite element analysis (FEA) in the explicit LS-DYNA code (Version 4.10) [54]. The modeling assumptions and boundary conditions are illustrated in Figure 5. To reduce computational time, a 4 mm section of the whole structure was used for simulation; prior studies have demonstrated that such conditions have no effect on the results [55,56]. The structure was positioned between two rigid plates, with the bottom plate fully constrained and the top plate allowed to move according to a specified condition. Both plates were meshed using eight-node hexahedron elements with steel properties. The auxetic structures were meshed using eight-node hexahedron elements with one central integration point. This element type is recommended for large deformations but requires hourglass control [57]. An element size of 0.5 mm was selected to ensure that the thinnest regions contained at least two elements through the wall thickness. Contact interactions were modeled using four penalty-based contact definitions to capture plate–specimen interactions, self-contact within the cellular structure, and interfacial contact in dual-material specimens. The friction between the surfaces was modeled using Coulomb’s formulation. The coefficients of friction were identified iteratively to determine the best outcomes compared to the experimental results. Two “Automatic_Surface_to_Surface” contacts were used for the plates and specimen contacts, with static and dynamic coefficients of friction set at $\mu_{s}$ = $\mu_{d}$ = 0.20. The self-contact of the specimen was simulated using “Automatic_Single_Surface” contact, with static and dynamic coefficients of friction set at $\mu_{s}$ = $\mu_{d}$ = 0.65. The interface of the dual-material specimen was simulated using “Automatic_Single_Surface_Tied” contact. A strain rate of 1 s^−1^ was adopted for the simulation considering the trade-off between computational efficiency and calculation cost. The increased strain rate compared to the experimental testing was justified because the simulations satisfied the quasi-static criteria [58,59]. The ratio of kinetic energy to internal energy was small (<5%); thus, the dynamic effects were negligible [60,61]. Despite these measures, the authors acknowledge that such a large strain rate difference may potentially affect the constitutive responses of the materials, the in-depth analysis of which is outside the scope of this work and can be undertaken in a future study. Based on the findings of previous studies [22,62], TPU95 was modeled using a simplified rubber model (MAT_181 in LS-DYNA) because it falls in the category of hyperelastic materials. PC was represented as an elastic–perfectly plastic material using a modified piecewise linear plasticity model (MAT_123 in LS-DYNA). The material properties were obtained from the technical data sheet provided by the manufacturer.

## 3. Results and Discussion

### 3.1. Quasi-Static Cyclic Response

**Figure 6:**
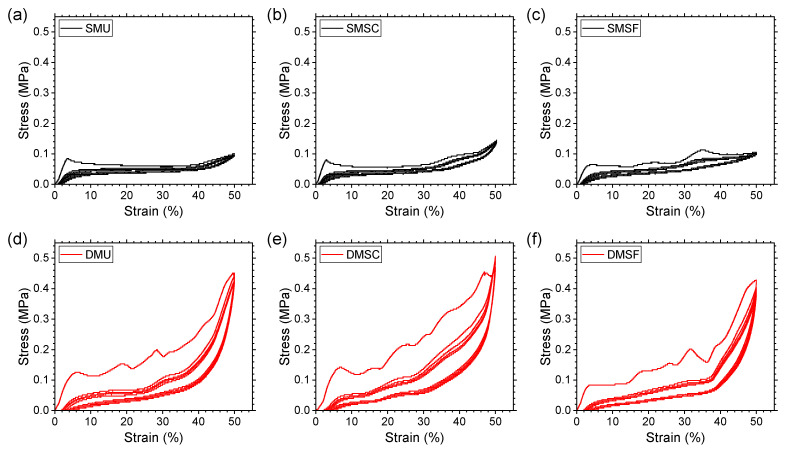
Stress–strain curves for single-material structures, (**a**) SMU, (**b**) SMSC, (**c**) SMSF; and dual-material structures, (**d**) DMU, (**e**) DMSC, (**f**) DMSF auxetic reentrant structures.

Compression tests were conducted for five repeated cycles for all three replicates in each specimen group. Figure 6 summarizes the stress–strain curves of the representative replicates for comparison purposes. Although the first cycle loading curves for all specimens showed three typical regions, linear elastic deformation, low stiffness plateau, and high stiffness densification, key differences can be observed in the curve shapes between single-material and dual-material specimens due to the existence of different deformation mechanisms (as discussed in Section 3.3 and Section 3.4). Single-material specimens showed a flatter plateau, whereas dual-material specimens showed a marked increase in the stress response over the plateau region. When compared pairwise within each structure type (i.e., uniform, strong core, and strong frame), the dual-material structures demonstrated higher yield stress and significantly higher densification stress compared to their single-material counterparts, suggesting better mechanical properties and energy absorption potential (as discussed in Section 3.2). Differences in compressive response also exist when compared among different structure types. For both single-material and dual-material structures, the strong core specimen showed a higher stress response across the whole compression region compared to uniform and strong frame structures.

TPU has been reported to demonstrate strain-softening behavior under repeated loading and unloading processes [63]. In this study, all specimens showed softening behavior, as evident from the stress–strain curves for the second cycle being much more compliant than those for the first cycle. Compared to single-material structures, dual-material structures showed a higher number of undulations along the plateau region in the first loading cycle and a relatively larger decrease in stress response in the second loading cycle. This phenomenon can be attributed to the existence of numerous TPU-PC interfaces in the dual-material structures. During the first loading cycle, interfaces or sections of interfaces with less than ideal layer-to-layer adhesion became weaker, which was not the case for single-material structures due to the lack of such interfaces. Regardless, reduced strain-softening behavior was observed for all specimens in subsequent cycles and the curves started to converge after the fourth cycle. This suggests that dual-material specimens demonstrated repeatable recovery behavior comparable to single-material specimens, which will be discussed later (Section 3.5).

### 3.2. Mechanical Properties and Energy Absorption Performance

**Figure 7:**
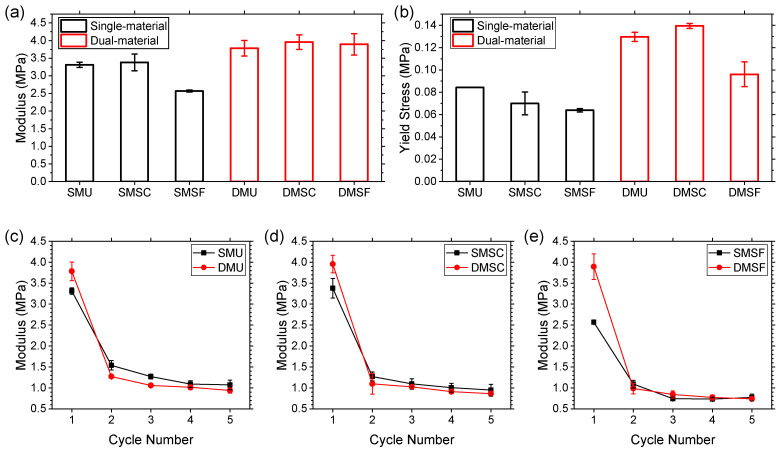
Comparison of (**a**) compressive modulus and (**b**) yield stress between the single- and dual-material structures, and (**c**–**e**) modulus profiles of all specimens.

The compressive modulus and yield stress of the reentrant structures are summarized in Figure 7. When comparing compressive modulus for the first compression cycle, dual-material structures demonstrated better performance with uniform, strong core, and strong frame structures, showing a 14%, 17%, and 52% increase, respectively (Figure 7a). Similar observations were made for yield stress, with a 54%, 99%, and 50% improvement for dual-material uniform, strong core, and strong frame structures, respectively (Figure 7b). Such enhanced properties could be attributed to the adoption of a dual-material design for the reentrant structures. It is worth noting that these enhancements were no longer noticeable in subsequent cycles once the structures became more compliant and the dual-material structures lost their less-than-ideal TPU-PC interfaces. Figure 7c–e show the modulus profiles of the uniform, strong core, and strong frame structures across five loading cycles. As mentioned earlier, a softening behavior was observed for all specimens, evident from a significant decrease in modulus in the second loading cycle. Following the second loading cycle, the decrease in modulus became stabilized and eventually converged in the fifth cycle. Once stabilized, the uniform structures showed higher modulus than the strong core, and the strong core showed higher modulus than the strong frame. The softening behavior can be attributed to several reasons [48,64]: (1) strain softening at molecular level, and (2) strain softening due to the breakdown and rearrangement of weak interfaces arising from layer-to-layer adhesion in the material extrusion process. Evidently, the structures do not immediately recover their original shape, thereby making it easier for them to be deformed in subsequent cycles.

**Figure 8:**
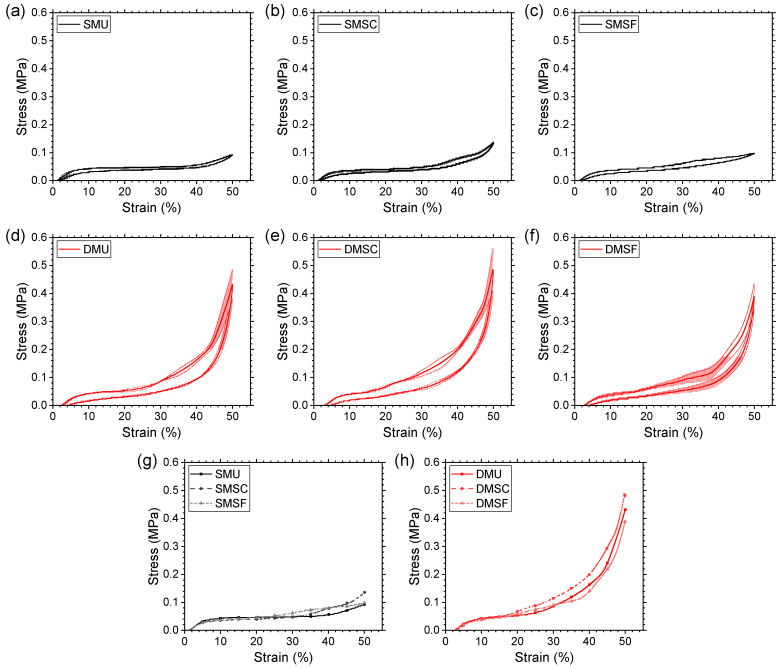
Stress–strain profiles of (**a**) SMU, (**b**) SMSC, (**c**) SMSF, (**d**) DMU, (**e**) DMSC, (**f**) DMSF, and comparison of stress–strain response for (**g**) single-material and (**h**) dual-material structures.

Based on the above-mentioned observations, the fifth compression cycle was used to compare the stress–strain response of all specimens (Figure 8). Each stress–strain profile (Figure 8a–f) includes the average response (solid line) of all three replicates for the corresponding specimen with the shaded area representing ±1 standard deviation from the average. All specimen groups showed good repeatability, as indicated by small variations throughout the curve, with single-material structures showing the least variations, and dual-material structures showing relatively larger stress variations in the high strain range. This can be attributed to the existence of weak TPU-PC interfaces in dual-material structures, and further weakening of such interfaces during multiple load/unload cycles resulted in larger variation among replicates at high strain regions. Despite these shortcomings, all dual-material specimen groups showed good recovery, similar to their single-material counterparts, with larger areas under the loading curve, areas enclosed by the loading/unloading hysteresis loop, and areas under the unloading curve, indicative of higher potential for energy absorption, dissipation, and release, respectively.

Figure 8g and Figure 8h compare the fifth loading cycles of the single- and dual-material structures, respectively. When compared within single-material structures, all specimen groups behaved similarly up to 25% compressive strain, beyond which the strong frame structure showed a higher stress response compared to the uniform structure, only to coincide again at 50% compressive strain. The strong core structure, on the other hand, showed a higher stress response compared to the uniform structure starting at 35% compressive strain, and continued to do so until 50% strain. In the case of dual-material structures, all specimen groups behaved similarly up to 15% compressive strain. In the range of 15–50% strain, the strong core structure had a higher stress response compared to the uniform structure. The strong frame, on the other hand, had a lower stress response compared to the uniform structure in the strain range of 32–50%. This indicates that there is no one best structure that outperforms the rest for both single-material and dual-material options.

**Figure 9:**
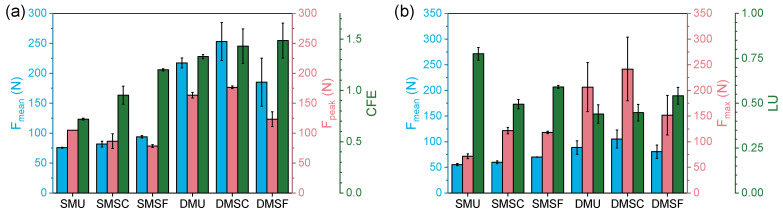
Comparison of (**a**) mean crushing force, peak crushing force, and crush force efficiency for the first loading cycle, and (**b**) mean crushing force, maximum crushing force, and load uniformity for the fifth loading cycle.

To provide a detailed assessment of the crashworthiness performance of the single- and dual-material structures, five crashworthiness metrics were evaluated for two different use case scenarios. If the cellular structures were to be used for one-time crushing applications (e.g., safety cushion, automotive protection, etc.), then the mean crushing force ($F_{mean}$), peak crushing force ($F_{peak}$), and crush force efficiency (CFE) can offer valuable information for appropriate selection (Figure 9a). On the other hand, if the structures were to be used for multiple loading applications (e.g., sportswear, seat cushions, etc.), then the mean crushing force ($F_{mean}$), maximum crushing force ($F_{max}$), and load uniformity (LU) can also provide signification insight (Figure 9b). Figure 9a summarizes the crashworthiness metrics of the single- and dual-material structures for the first loading cycle. Clearly, dual-material structures showed relatively higher $F_{mean}$ and $F_{peak}$, with 287%, 310%, 196% and 155%, 203%, 157% improvement for uniform, strong core, and strong frame structures, respectively. This suggests that dual-material structures possess significantly better load-bearing capabilities and higher potential for energy absorption when it comes to one-time crushing applications. In terms of CFE, however, single-material structures represented values closer to 1 compared to dual-material structures, with SMSC (CFE = 0.95) performing the best out of all six designs. This can be attributed to the relatively flatter stress plateaus of single-material structures, as discussed earlier (Figure 6 and Section 3.1). Figure 9b highlights the crashworthiness metrics of the single- and dual-material structures for the fifth loading cycle. As stated in Equation (4), $F_{max}$ was used instead of $F_{peak}$ to define LU, as initial peak force appeared only in the first loading cycle (Figure 6). As can be seen from Figure 9b, dual-material structures showed relatively higher $F_{mean}$ and $F_{max}$, with 160%, 175%, 115% and 288%, 198%, 128% improvement for uniform, strong core, and strong frame structures, respectively. Again, this suggests that dual-material structures possess better load-bearing capabilities and can serve as excellent energy-absorbing structures when it comes to repeated loading applications. However, the single-material structures showed better LU compared to the dual-material structures, with SMU showing the most consistent stress response (LU = 0.78) among all six designs, which is again attributed to the relatively flatter stress response under repeated loading conditions (Figure 8).

**Figure 10:**
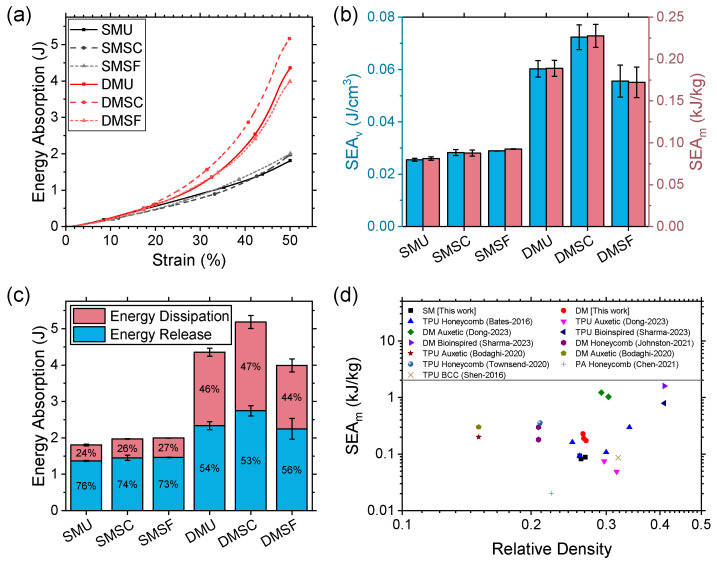
Comparison of (**a**) energy absorption, (**b**) volumetric and gravimetric specific energy, (**c**) and energy dissipation and release for single- and dual-material structures; (**d**) specific energy absorption with different energy-absorbing structures: TPU honeycomb [63,66], TPU and DM auxetic [17,35], TPU and DM bioinspired [65], DM honeycomb [36], PA honeycomb [67], and TPU BCC [68].

The energy absorption characteristics of the reentrant structures are summarized in Figure 10 for the fifth loading cycle. The energy absorption of the structures increased linearly with an increase in compressive strain up to 20% (Figure 10a). However, as indicated earlier, dual-material structures showed exponential growth in energy absorption beyond 20% strain, whereas single-material structures showed a linear increase. This is due to the marked increase in the stress–strain curve of the dual-material structures beyond 20% compressive strain (Figure 8). Evidently, all dual-material structures had a segmented plateau with increasing slopes. While SMSC and SMSF also had segmented plateau, owing to their gradient profiles, the slopes of dual-material structures were much steeper than the single-material ones, hence the exponential growth in energy absorption compared to the linear increase. It is worth noting that single-material structures showed almost similar energy absorption profiles irrespective of gradient profiles. However, in the case of dual-material structures, the strong core structure showed significantly higher energy absorption performance owing to its higher mean, peak, and maximum crushing force (Figure 9). Interestingly, all structures investigated in this study had similar relative densities (Table 4); therefore, increased energy absorption performance did not come at the cost of increased mass. Rather, the strategic placement of rigid and flexible materials in the dual-material structures played a key role in governing the deformation mechanics (as discussed in Section 3.4).

Volumetric and gravimetric energy absorption, also known as specific energy, are important metrics for characterizing energy absorption performance, since these are used to normalize the effect of volume and mass when comparing materials and structures with varied densities. Energy absorption per unit volume (SEA_v_) offers valuable insights when selecting structures for applications with space constraints, whereas energy absorption per unit mass (SEA_m_) is important for applications with weight constraints. In this study, the structures were designed to have the least variation in their densities for easier comparison. Therefore, as seen in Figure 10b, relatively similar patterns were observed for both SEA_v_ and SEA_m_. All dual-material structures showed superior specific energy absorption, with approximately 234%, 260%, and 189% improvement for uniform, strong core, and strong frame structures, respectively.

All reentrant structures were capable of relatively higher energy release compared to energy dissipation, as depicted in Figure 10c. This indicates that these structures demonstrated better recovery behavior than damping potential. Energy release comprised approximately 75% of the total energy for single-material structures, whereas it was around 55% of the total energy for dual-material structures. The higher proportion of energy release in the case of single-material structures is attributed to the hyperelastic nature of the parent TPU material, which can store considerable elastic energy. In the case of dual-material structures, rigid PC contributed more toward energy dissipation than energy release; therefore, the elastic energy storage capability of TPU was reduced. However, in all cases, dual-material structures showed higher absolute values of energy dissipation and energy release, with 464%, 470%, 328% and 170%, 189%, 153% improvements for uniform, strong core, and strong frame structures, respectively.

Figure 10d compares the specific energy absorption of single- and dual-material structures with other structures reported previously [35,36,39,63,65,66,67,68]. While the single-material structures performed toward the lower end of the spectrum and were similar to other single-material structures, dual-material structures were competitive with other dual-material structures. Overall, dual-material structures showed higher specific energy absorption compared to single-material structures. This indicates that applications that require higher energy absorption performance can benefit from a lightweight dual-material design.

### 3.3. Finite Element Analysis Validation

**Figure 11:**
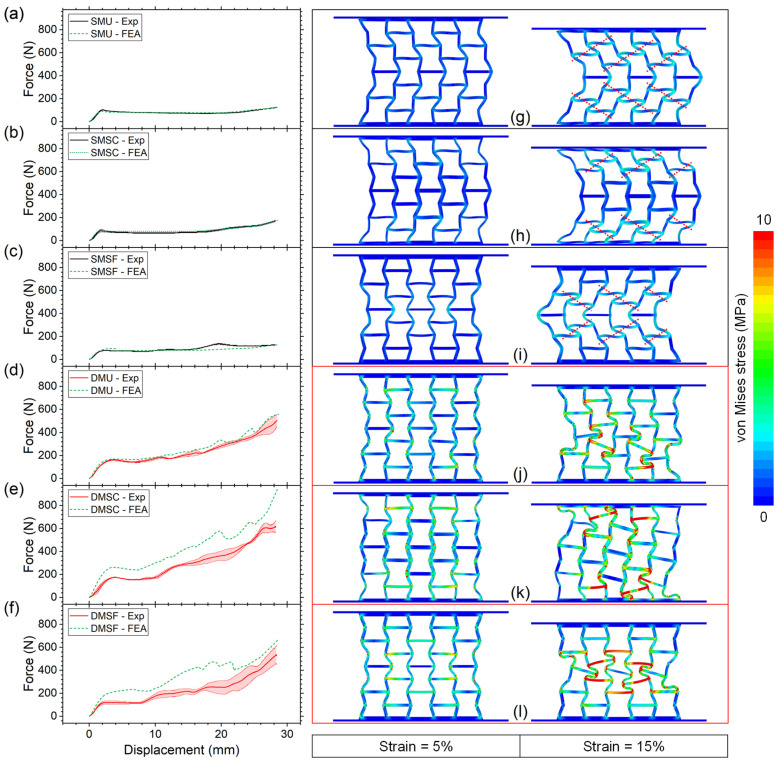
(**a**–**f**) Comparison of experimental and simulation force–displacement curves and (**g**–**l**) stress distribution at strains of 5% and 15% for single-material and dual-material structures.

The numerical simulation results were validated with the experimental results to verify the material models and boundary conditions defined for the finite element analysis. Figure 11a–f show the comparison of the force–displacement curves between the experimental results and the numerical simulations. The predicted curves were found to be very similar to the experimental curves for all three single-material structures. For dual-material structures, the predicted curve showed slightly higher force responses for uniform structures but significantly higher responses for strong core and strong frame structures in comparison to the experimental results. The reason for such differences in force response is not readily clear. However, a possible explanation is that unlike single-material structures, a large number of material-to-material interfaces (rigid and flexible) exist in dual-material structures. The failure criteria of these interfaces were not modeled into the finite element analysis as this was outside of the scope of this study and requires comprehensive study focusing on interface mechanics. Regardless, it can be assumed that the interfaces for the specimen were not as strong as the simulated interfaces modeled with tied contact. Therefore, given the large number of interfaces with smaller surface-to-surface contact in strong core and strong frame structures, these structures showed significantly higher force responses in comparison to uniform structure.

Under compressive loading, the single-material and dual-material auxetic structures responded differently as shown in Figure 11g–l. At 5% compressive strain, all but one (strong frame) single-material structure protruded symmetrically to the right side. This was due to the hyperelastic nature of TPU, which resulted in elastic instability. Similar deformation behavior has been reported previously for uniform-thickness auxetic structures made of elastomeric or flexible materials [25,39,43,44,69,70]. The strong frame structure deformed inwards owing to the relatively thinner cell walls in the core cells, which indicates higher potential for a negative Poisson’s ratio effect. However, with increasing compressive strain, the instability set in, causing the strong frame structure to also protrude to one side. At this stage, all single-material structures showed noticeable elastic bending of the horizontal and inclined cell walls, causing some of these walls to come closer to each other, forming symmetrical compaction bands (highlighted with red dotted line). In the case of SMU, compaction bands appeared throughout the whole structure, whereas the bands were observed across top/bottom row cells and middle row cells for SMSC and SMSF, respectively. Consequently, the cell walls falling across the compaction bands experienced the majority of the compressive stress.

In contrast to single-material structures, all dual-material structures deformed inward at 5% compressive strain, resembling common deformation behavior observed in reentrant auxetic structures built from rigid thermoplastics [39,40]. While DMU showed stress concentration throughout the whole structure, DMSC and DMSF showed localized stress concentrations around frame cells and core cells, respectively, owing to their thinner cell wall thickness, as shown in Figure 11j–l. Unlike single-material structures which showed outward protrusion due to lack of lateral stiffness, clearly, the rigid horizontal cell walls in dual-material structures resisted excessive bending, which effectively prevented the structures from protruding on either side. As a result, with increasing compressive strain, dual-material structures continued to deform inward with varied deformation characteristics. At 15% compressive strain, DMU started to show diagonal collapse of its cells, whereas in the case of DMSC and DMSF, the frame cells and core cells collapsed, respectively, through significant elastic deformation of the TPU cell walls. These results indicate strong potential for partial or full recovery of the dual-material structures.

### 3.4. Deformation Patterns and Poisson’s Ratio

**Figure 12:**
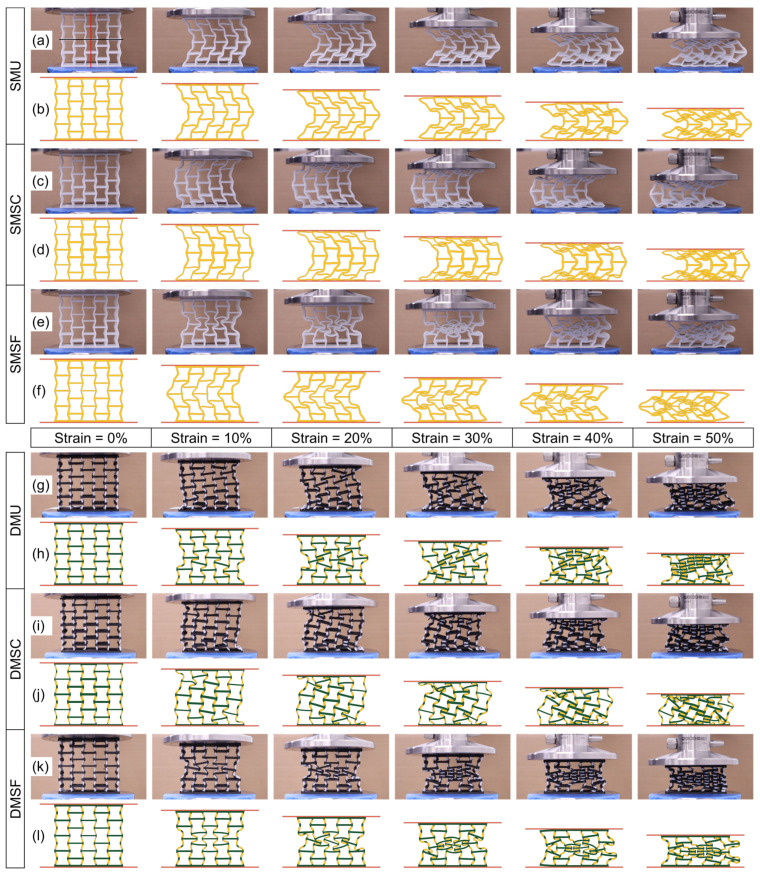
Comparison of (**a**,**c**,**e**,**g**,**i**,**k**) experimental and (**b**,**d**,**f**,**h**,**j**,**l**) simulated deformation patterns of single- and dual-material structures.

The compressive behavior of the single- and dual-material structures were recorded to understand the local and global deformation patterns with increasing strain levels. Figure 12 shows the deformation patterns of experimental tests (video images) and their corresponding numerical simulation results at selected stages of the deformation process. The simulated deformation patterns showed good agreement with the observed experimental results. Given the imaginary horizontal (black) and vertical (red) planes of symmetry in Figure 12a, both SMU and SMSC gradually deformed, with horizontal symmetry and vertical asymmetry governed by elastic instability. The direction of the protrusion was found to be random. Regardless of the direction of protrusion, the simulated patterns very closely resembled the experimental observations. As for the SMSF structure (Figure 12e), owing to the thinner core cell wall thickness in comparison to that of the frame cell, the elastic instability was not significant enough to cause the protrusion. Therefore, the structure deformed symmetrically with respect to both horizontal and vertical planes, representative of ideal negative Poisson’s ratio behavior. While SMU and SMSC continued to protrude outward from start to finish, SMSF collapsed inward to form a denser core and eventually protruded outward due to elastic instability. This transition occurred at approximately 35% compressive strain, which resulted in a peak in the stress response (Figure 6c). The simulated deformation patterns of the SMSF deviated from those in the experimental results. This could be due to the differences arising from the microscopic variation present in the experimental specimens in the layer-by-layer building process. The simulated parts were assumed to be made of homogenous material, which is the most commonly used methodology for specimens built using the material extrusion additive manufacturing process [34,35,37,38].

The deformation patterns of the dual-material structures revealed gradual inward bending of the cells with varying levels of localized collapse owing to different cell wall thicknesses. Unlike single-material structures, the lack of outward protrusion in dual-material structures indicates that the inclusion of the rigid walls in the dual-material design effectively minimized elastic instability. This can potentially improve the desired negative Poisson’s ratio effect. Additionally, the inward collapse of the structure resulted in a significantly higher stress response in dual-material structures (Figure 6). Both DMU and DMSC showed diagonal collapse with increasing compressive strain, indicating that the strong-core cells had a minimal effect in governing the collapse mechanism. In the case of DMU, the cells collapsed randomly, whereas the frame cells were found to collapse first, followed by the partial collapse of the core cells in DMSC. On the contrary, DMSF showed similar collapse behavior as SMSF, where the thinner wall core cells collapsed first, promoting the stable inward global collapse of the structure, followed by the partial collapse of the frame cells.

**Figure 13:**
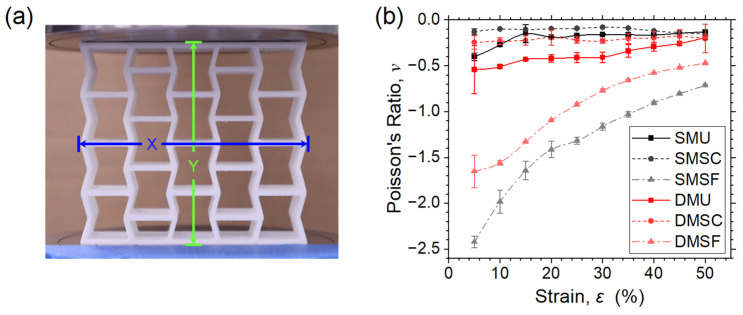
(**a**) Section used for measuring strain along the X direction and (**b**) comparison of Poisson’s ratio for reentrant structures.

Figure 13 illustrates the Poisson’s ratio of both single-material and dual-material structures during the compression process. The image frames at a 5% strain increment from the recorded deformation process were used to identify the history of the Poisson’s ratio of the structures (Figure 13b). The strain across the transverse direction was measured along the middle plane and longitudinal direction using the timestamp of the corresponding image frame, as shown in Figure 13a. Across the whole compression strain range, Poisson’s ratio stayed negative for all the structures with varied degrees of strain dependency. Both strong core structures showed almost no strain dependency, whereas uniform structures showed relatively more dependency, followed by strong dependency in the case of strong frame structures. The same can be concluded for the negative Poisson’s ratio in terms of the strongest to weakest auxetic effect in the following order: strong frame ≫ uniform > strong core. In the case of the strong core structures, the Poisson’s ratio of SMSC stayed almost stable at approximately −0.11, whereas for DMSC, it was approximately −0.25 at the beginning and then increased gradually with the longitudinal strain to approximately −0.20. Both uniform structures showed a reduced negative Poisson’s ratio at the beginning compared to their strong core counterparts, with SMU and DMU valued at approximately −0.4 and −0.54, respectively. As the compressive strain increased, Poisson’s ratio also increased gradually to −0.13 and −0.19, respectively. For both strong core and uniform structures, dual-material structures showed improved Poisson’s ratio effect compared to single-material structures, which highlights the benefit of dual-material design. However, in the case of strong frame structures, the opposite was observed. SMSF started with the lowest Poisson’s ratio of approximately −2.42, whereas DMSF started with a Poisson’s ratio of −1.65. With increasing compressive strain, the Poisson’s ratios increased rapidly to approximately −0.72 and −0.47 for SMSF and DMSF, respectively. This indicates that a strong frame structure design can significantly improve the negative Poisson’s ratio effect in reentrant auxetic structures.

### 3.5. Short-Term Recovery and Long-Term Durability

**Figure 14:**
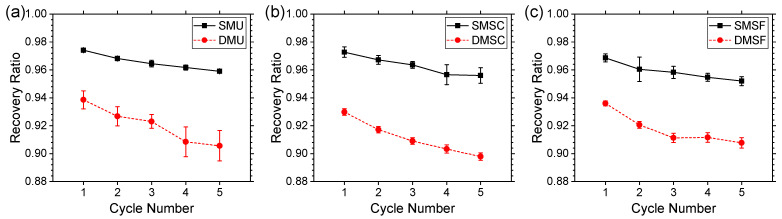
Comparison of recovery ratio for single-material and dual-material structures: (**a**) uniform, (**b**) strong core, and (**c**) strong frame.

The extent to which the single-material and dual-material structures recovered during the cyclic compressive loading was measured using the recovery ratio. Figure 14 depicts the differences in recovery ratio between single-material and dual-material structures. For all structures, the recovery ratio decreased in the subsequent compression cycle. The dual-material structures demonstrated relatively lower recovery performance compared to their single-material counterparts. At the end of the first cycle, the recovery ratio of the dual-material structures was approximately 3.8% lower compared to the single-material structures. It further increased to 5.4% at the end of the fifth cycle. Such a trade-off in recovery performance in the return of the enhanced force response in the dual-material structures was expected as they consist of interfaces where two materials join with each other, unlike single-material structures. The lack of these interfaces in the single-material structures allowed them to deform and recover without any permanent damage to the joints of rotation. The dual-material structures, on the other hand, experienced permanent delamination in some of the dual-material interfaces, causing it to lose its recovery performance to some extent. Regardless, on average, single- and dual-material structures showed 95.5% and 90% recovery ratios at the end of fifth cycle, demonstrating excellent recovery performance, which is potentially suitable for repeated loading applications. Upon full recovery of the structures after 24 h, the single- and dual-material structures showed approximately 99% and 96% recovery ratios, respectively, which is indicative of the reversible nature of the proposed gradient and dual-material designs.

**Figure 15:**
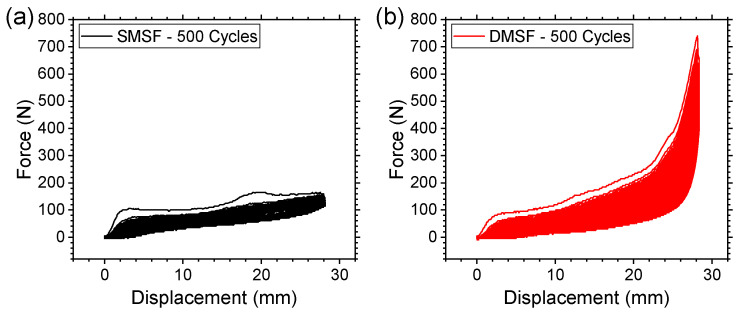
Comparison of long-term durability for strong frame (**a**) single-material and (**b**) dual-material structures.

Considering the overall performance of the single-material and dual-material structures in terms of mechanical properties, deformation mechanics, and negative Poisson’s ratio effect discussed thus far, the strong frame structures were subjected to a 500-cycle compression test to assess their long-term durability. Figure 15 depicts the force–displacement curves of the durability tests. Both SMSF and DMSF were found to be able to withstand 500 cycles of compression without any noticeable damage or structural failure. While both structures required multiple cycles of compression to stabilize (i.e., achieve compliance), SMSF stabilized sooner than DMSF, as expected. It is worth noting that DMSF continued to demonstrate significantly enhanced force response up until the 500th cycle of compression. Such enhanced performance is attributed to the dual-material design proposed in this study, which allowed DMSF to withstand a force that is as much as 207% higher than that of SMSF. Given the excellent energy absorption properties of the dual-material structures, with comparable shape recovery and long-term durability relative to single-material structures, it is possible to conclude that the density-graded dual-material design approach addresses some of the limitations of conventional reentrant structures without any significant performance trade-off.

## 4. Conclusions

A new reentrant auxetic structure was proposed utilizing the synergy between in-plane density gradient design and dual-material extrusion technology. Two density gradient reentrant structures along with their equivalent density regular reentrant auxetic counterparts were designed, fabricated in single-material and dual-material modes, and tested under in-plane quasi-static compression. The effects of gradient and material on the compressive response, deformation patterns, and energy absorption characteristics were investigated. The finite element analysis results were validated against the experimental results. The Poisson’s ratio, along with short- and long-term durability, was also reported. The results indicate that dual-material structures exhibited higher yield stress compared to single-material structures, with 54%, 99%, and 50% improvement for uniform, strong core, and strong frame structures, respectively. Though dual-material structures demonstrated higher modulus in the initial compression cycle, such an improvement was not permanent, and the modulus became similar to single-material structures upon reaching compliance. Dual-material structures showed significantly higher $F_{mean}$ and $F_{peak}$, with 287%, 310%, and 196% and 155%, 203%, and 157% improvement for uniform, strong core, and strong frame structures, respectively, for the first loading cycle. Furthermore, in the case of the fifth loading cycle, similarly higher $F_{mean}$ and $F_{max}$ were observed, with 160%, 175%, and 115% and 288%, 198%, and 128% improvement for uniform, strong core, and strong frame structures, respectively. Single-material structures, on the other hand, showed better LU and CFE. Owing to the significant increase in energy absorption beyond 20% compressive strain, dual-material structures exhibited superior specific energy absorption, with approximately 234%, 260%, and 189% improvement for uniform, strong core, and strong frame structures, respectively. Excellent energy dissipation and energy release were also observed, with 464%, 470%, and 328% and 170%, 189%, and 153% improvements, respectively. Unlike single-material structures, the inclusion of rigid walls in the dual-material design reduced excessive bending, which effectively minimized elastic instability, and therefore prevented the structures from protruding on either side. The strong frame design enhanced negative Poisson’s ratio effect of the reentrant auxetic structure. Both single- and dual-material structures excelled in repeated loading applications, with 95% and 90% recovery ratios. Dual-material structures also showed long-term durability similar to single-material structures, with consistently higher force responses. Through the strategic placement of rigid and flexible materials in a dual-material reentrant auxetic structure, along with two gradient schemes, this work has demonstrated a new method to overcome the limitations of reentrant auxetic structure design, potentially making it an excellent choice as a lightweight energy-absorbing structure with recovery capabilities.

## Figures and Tables

**Table 1:** Design parameters and geometric specifications used for the auxetic structures.

*L* (mm)	*t* (mm)	*θ* (°)	*h* (mm)	*a* (mm)	*b* (mm)
14	variable	75	7.25	1	t/2

**Table 2:** Design parameters, geometric specifications, and theoretical relative densities of the honeycombs.

Specimen	Core Wall Thickness, $t_{c}$ (mm)	Middle Wall Thickness, $t_{m}$ (mm)	Frame Wall Thickness, $t_{f}$ (mm)	Length, L (mm)	Width, W (mm)	Height, H (mm)	Relative Density, $\rho_{r}$
SMU	1.38	1.38	1.38	63.92	20	57.40	0.255
SMSC	1	1.3	1.6	63.53	20	57.02	0.253
SMSF	2	1.5	1	63.53	20	57.02	0.255
DMU	1.38	1.38	1.38	63.92	20	57.40	0.255
DMSC	1	1.3	1.6	63.53	20	57.02	0.253
DMSF	2	1.5	1	63.53	20	57.02	0.254

**Table 3:** Process parameters used for 3D printing.

Parameter	Single-Material	Dual-Material
Material	TPU	TPU/PC
Nozzle temperature (°C)	215	215/255
Bed temperature (°C)	70	100
Nozzle diameter (mm)	0.4	
Infill density (%)	100	
Infill pattern	Grid	
Layer height (mm)	0.15	
Wall line count	3	
Printing speed (mm/s)	20	

**Table 4:** Dimensional properties of the 3D printed auxetic structures.

Specimen	Length, L (mm)	Width, W (mm)	Height, H (mm)	Mass, m (g)	Relative Density, $\rho_{r}$
SMU	63.32 ± 0.02	19.68 ± 0.05	56.78 ± 0.04	22.23 ± 0.13	0.262 ± 0.001
SMSC	62.96 ± 0.11	19.65 ± 0.06	56.42 ± 0.02	22.47 ± 0.13	0.268 ± 0.000
SMSF	62.48 ± 0.07	19.62 ± 0.13	56.29 ± 0.03	21.57 ± 0.13	0.260 ± 0.001
DMU	63.44 ± 0.20	20.03 ± 0.04	56.88 ± 0.09	23.07 ± 0.05	0.266 ± 0.001
DMSC	63.28 ± 0.04	20.03 ± 0.07	56.55 ± 0.02	22.77 ± 0.05	0.265 ± 0.002
DMSF	63.26 ± 0.02	20.00 ± 0.04	56.74 ± 0.03	23.17 ± 0.17	0.269 ± 0.002

## Data Availability

The original contributions presented in this study are included in the article. Further inquiries can be directed to the corresponding author.
